# Complete mitochondrial genome of *Dibranchus japonicus* (Actinopterygii, Lophiiformes, Ogcocephalidae), from the West Pacific Ocean

**DOI:** 10.1080/23802359.2021.1915203

**Published:** 2021-07-06

**Authors:** Mei-Ling Ge, Wen-Ge Shi, Yi-Xuan Li, Zhong Li, Xue-Lei Zhang, Qin-Zeng Xu

**Affiliations:** a College of Environmental Science and Engineering, Ocean University of China, Qingdao, China; b Key Laboratory of Marine Eco-Environmental Science and Technology, First Institute of Oceanography, Ministry of Natural Resources, Qingdao, China; c Department of Biology, Faculty of Science, Hong Kong Baptist University, Kowloon Tong, Hong Kong, China

**Keywords:** Ogcocephalidae, *Dibranchus japonicus*, mitochondrial genome, phylogeny

## Abstract

*Dibranchus japonicus* is a benthic fish living in the deep Pacific Ocean. Here, we described the complete mitochondrial genome of this species, with the sequences about 17,233 bp in length, containing 13 protein-coding genes (PCGs), 22 tRNAs, and two rRNAs. The gene arrangement of this species was identical with others from family Ogcocephalidae. The content of GC and AT for *D. japonicus* was 45.41% and 54.59%, respectively. Phylogenetic analysis, based on 13 PCGs and two rRNA genes, revealed the close relationship between *D. japonicus* and other species of Ogcocephalidae, which was consistent with the morphology.

*Dibranchus japonicus* Amaoka & Toyoshima, 1981 belongs to class Actinopterygii, order Lophiiformes, family Ogcocephalidae, genus *Dibranchus*. That is a kind of small benthic fishes in the continental, which mainly distributes in the Pacific Ocean from New Zealand to the Aleutian Islands (Fishbase 2020). Most species of Ogcocephalidae are deep-sea fishes, feeding on some small fishes and benthic invertebrates (Bradbury 1999; Cruz-Acevedo et al. 2019). The genus *Dibranchus* is an typical inhabitant of deeper waters (Amaoka and Toyoshima, 1981), having at least 14 valid species described to date (Worms 2020). For the family Ogcocephalidae, only four mitochondrial genomes from the genus *Halieutaea*, *Malthopsis*, *Zalieutes*, and *Coelophrys* were reported. Herein, the complete mitochondrial genome of *D. japonicus* was first sequenced to deepen the comprehension of the genetic phylogenetic relationship for Ogocephalidae.

A specimen of *D. japonicus* was collected from the Pacific Ocean (139°54′E, 9°43′N) and deposited in Key Laboratory of Marine Eco-Environmental Science and Technology, First Institute of Oceanography, Ministry of Natural Resources (No. FIO-PAC-CJ0901). The mitochondrial genome sequence of *D. japonicus* was examined on the Illumina HiSeq 2500 Sequencing Platform (Illumina, Hayward, CA) by Novogene Corporation (Beijing, China). The sequences were assembled using SPAdes 3.6.1 (Bankevich et al. 2012) and blast against with other species from Ogcocephalidae in GenBank to query mitochondrial genomic fragments. The gaps were filled by Price (Ruby et al. 2013) and MITObim v1.8 (Hahn et al. 2013). The resultant reads were obtained from bowtie2 (Langmead and Salzberg 2012) and reassembled. Bandage (Wick et al. 2015) was used to verify the circular structure of the mitochondrial genome.

The complete mitogenome genome of *D. japonicus* (GenBank accession: MW080645) was 17,233 bp in length, containing 13 protein-coding genes (PCGs), two rRNA genes, 22 tRNA genes, and one control region. The overall nucleotide compositions were 27.9% A, 28.0% C, 17.4% G, and 26.7% T. The gene order was in accordance with other species from the Ogcocephalidae, indicating that the gene order was conserved in this family.

A maximum-likelihood tree was constructed by IQTREE (Nguyen et al. 2015) using the dataset with 13 PCGs and two rRNAs. Fifteen species from the same order with *D. japonicus* (Lophiiformes) were chosen with three species of other orders as outgroups. The nucleotide partition models of each gene were determined by ModelFinder (Kalyaanamoorthy et al. 2017). As the phylogenetic analysis results showed, *D. japonicus* and others from Ogcocephalidae formed a branch, and then gathered with other families of the order Lophiiformes. The genetic phylogenetic results were consistent with the morphological phylogeny. Furthermore, *D. japonicus* was mostly closed to *Coelophrys brevicaudata* comparing with other species from Ogcocephalidae. This mitochondrial genome analysis will provide the foundation for further phylogeny study on the genus *Dibranchus*. Further studies on the mitochondrial genome of *Dibranchus* were needed to explore the relationship of this genus (Figure 1).

**Figure 1.**
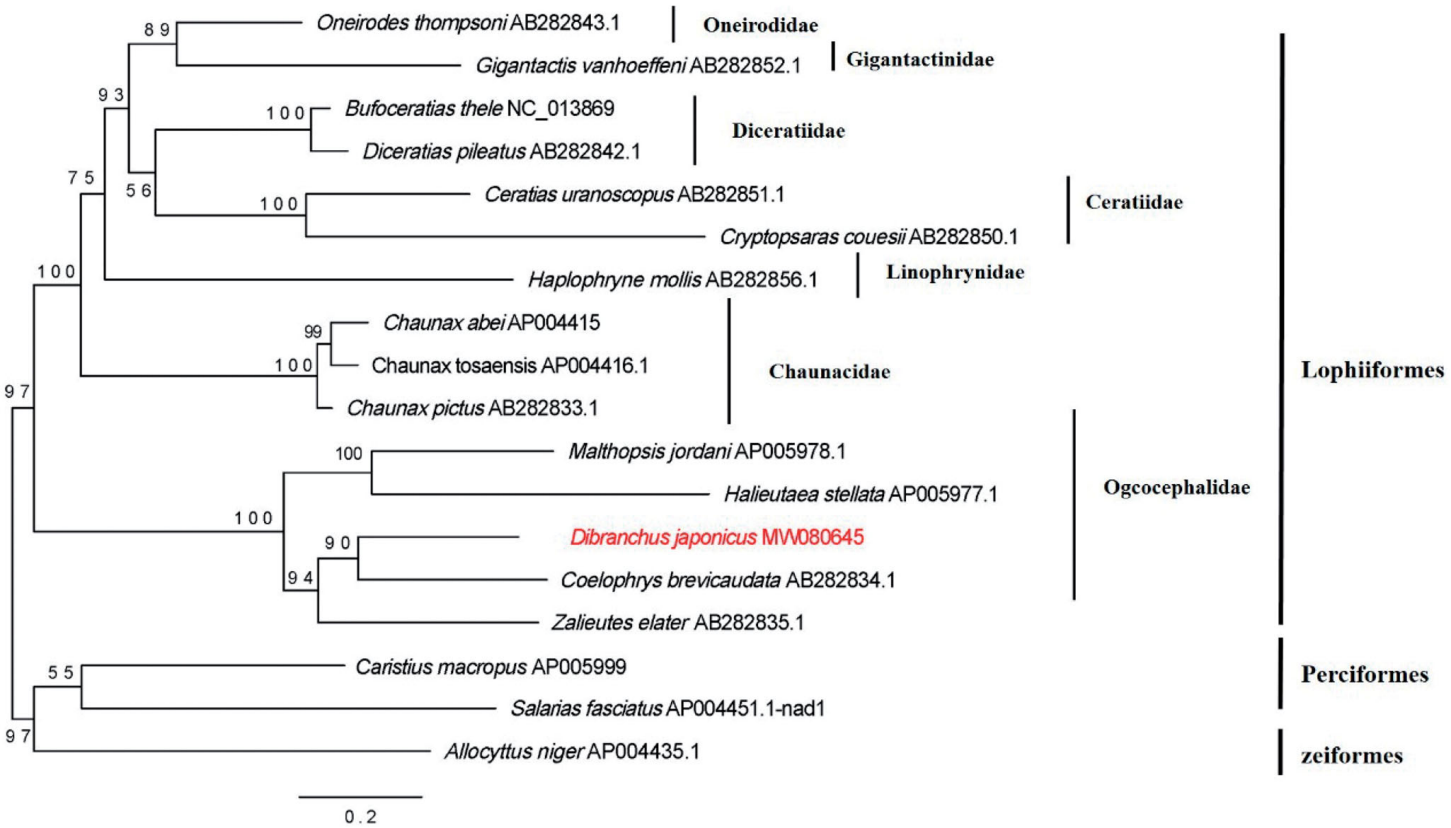
The maximum-likelihood tree of the Ogcocephalidae based on 13 PCGs and two rRNAs. Number at branch represents bootstrap probability.

## Data Availability

The genome sequence data that support the findings of this study are openly available in GenBank of NCBI at https://www.ncbi.nlm.nih.gov/nuccore/ under the accession no. MW080645. The associated BioProject, SRA, and Bio-Sample numbers are PRJNA685319, SRR13258663, and SAMN17080907, respectively.
